# Supplementary material for: ENSO modulates wildfire activity in China
Source: Nat Commun. 2021 Mar 19;12:1764. doi: 10.1038/s41467-021-21988-6 (PMC7979797; doi:10.1038/s41467-021-21988-6)
Supplement: Supplementary file 1 — Supplementary Information [file 41467_2021_21988_MOESM1_ESM.docx]

**Supplementary Information**

for

**ENSO modulates wildfire activity in China**

Keyan Fang^1,2, &,*^, Qichao Yao^3, 4,&^, Zhengtang Guo^5,6^, Ben Zheng^7^, Jianhua Du^8^, Fangzhong Qi^4^, Ping Yan^3^, Jie Li^3^, Tinghai Ou^2^, Jane Liu^1,9^, Maosheng He^10^,

Valerie Trouet^11,*^

^&^ K. F. and Q. Y. contributed equally to this work.

^*^ To whom correspondence should be addressed: kujanfang@gmail.com and trouet@ltrr.arizona.edu

**This PDF file includes:**

Supplementary Information

Supplementary Figure 1-22

Supplementary Table 1-2

Supplementary References

**The wildfire monitoring system**

The data of the Wildfire Atlas of China (WFAC) were derived from the Forest Fire Prevention and Monitoring Information Center of China (FFPMIC). The FFPMIC includes a data analysis center and five ground stations to receive all polar orbit images from 6 series of around ten satellites (number of satellites varies through time) to cover the whole country for over ten times a day. The 5 satellite ground stations are at Beijing (39.95°N, 116.42°E), Kunming (25.06°N, 102.69°E) in southwestern China, Harbin (45.17°N, 127.31°E) in northeastern China, Urumqi (43.31°N, 87.27°E) in northwestern China, and Sanya city (18.17°N, 109.43°E) in southeastern China. The ground stations have 5.4 m caliper aerials to receive signals of the X (7750 MHz–8500 MHz) and L (1670 MHz–1710 MHz) frequency bands with elevation angles over 5° and 3°, respectively. The 5 ground stations exchange their satellite images via the data sharing cloud platform for data interpretation. This multiple node monitoring system receives data from multiple satellites and crosschecks the data from these satellites, avoiding for the lack of monitoring data due to the failure of the reception of a satellite image.

The ground stations received all the images from polar orbit satellites, including 6 satellites series of about ten satellites, with the number of satellites varying through time (Supplementary Table 2). They include satellite series from the National Oceanic and Atmospheric Administration (NOAA) satellite series (NOAA 15/16/17/18/19; now mainly from the NOAA18/19). The instruments carried by the satellite series are the Advanced Very High Resolution Radiometer (AVHRR). The second satellite series is from the NASA Earth Observing System (EOS), including Terra and Aqua. The satellite-borne instruments are the Moderate Resolution Imaging Spectroradiometer (MODIS). The third satellite series is the FengYun-2 (FY-2) and FY-3 satellite series (now mainly from FY-3). The satellite-borne instruments in FY are Visible and Infrared Radiometer (VIRR) from FY3 A/B/C/D and Medium Resolution Spectral Imager (MERSI) from FY3 A/B/D. The fourth is from the Meteorological Operational (METOP) satellite series. They carry instruments of the AVHRR. The fifth satellite series are the Suomi National Polar-orbiting Partnership (Suomi-NPP), which carries instruments of the Visible Infrared Imaging Radiometer Suite (VIIRS). The last satellite series are from the Joint Polar Satellite System (JPSS) satellites, which carry instruments of the VIIRS.

The ground station first makes the satellite data receiving plan based on the satellite attitude parameters and then controls the antenna to be directed to the satellite to receive the L-band and X-band image signals of the satellite broadcast. The transmission time of the entire track image data is no more than 5 minutes. The satellite channel system is responsible for the frequency conversion, filtering and decoding of the satellite signal according to the instructions of the station management system. The incoming fast vision system converts the digital signals by the decoder into zero-level image data, and quickly previews the image during the receiving process.

The data interpretation center performs quality inspection, radiation correction, positioning, image enhancement and projection processing on the zero-level image data to form first-level image data with extracted wildfire information. The preprocessing time of the entire track L-band data is less than 2 minutes, and the whole track X-band data is less than 15 minutes. The satellite data storage management system stores the original satellite images and forest fire monitoring results, providing services such as query, retrieval and download. The first-level wildfire monitoring results are shared with the forest fire prevention departments.

**Methods on fire identification from satellite images**

1. Multi-band image synthesis

During the daytime, the visible channels of the satellite are displayed in blue and green colors. The middle and infrared channel is in red for pseudo color synthesis to produce wildfire monitoring images. It aims to ensure the vegetation is green, bare soil and desert are yellow or brown, farmland grassland is light green, water bodies are blue, clouds are white, the open fires are bright red and dying fire are dark red. At night, the images are displayed by two infrared channels with a wavelength of 10μm. A variety of image stretching and enhancement functions are used to adjust the image contrast and brightness.

2. Geometric positioning

Multiple modes are used for positionings, such as one-point positioning, multi-point positioning, and geographic information registration. The system has the information of the projection of the image data that allow the image to be positioned, enhanced, superimposed with geographic information, and forest fire monitoring as the original image. The background information to be added includes the latitude, longitudes, the administrative division borders and names, and the 1:25 million national water system maps.

3. Identifying the wildfire hotspots

The wildfires are identified using the fire identification model provided by NASA ^1^. The abnormal hotspots are identified after the seasonal adjustments of the seasons and the solar height angles. The extraction of hotspot information filters out the influence of other heating sources such as conventional thermal anomalies and solar reflection points.

The hotspots are marked according to the regulations and requirements of "national satellite forest fire monitoring management regulations" issued by the Office of National Forest Fire Protection Command and the "standards for compiling satellite forest fire monitoring results" issued by the Forestry Standard.

**Classification of the WFAC into 10 groups**

Classifications of the WFAC into 10 regions are detailed below:

1. Southern China (SOC) group includes a square area of 19.5-23.5°N 106.5-118.5°E and a neighboring area with three grids from 98.5-102.5°E at 21.5°N. The fire grid of 23.5°N 106.5°E shows the second highest loading on the SOC pattern, but its loading is even high for the neighboring SW pattern, so we classified it to the SWC pattern. The SOC includes 11 grids with the highest loading over factor 1 and 3 grids with the second highest loadings over factor 1 but surrounded by the fire grids with the highest loadings.

2. Southwestern China (SWC) pattern includes fire grids of a square area of 23.5-27.5°N 98.5-106.5°E. All the 15 grids in the area have the highest loadings over the factor 3.

3 Southeastern China (SEC) pattern includes a square area of 25.5-27.5°N 108.5-120.5°E. This pattern has ten grids showing the highest loadings over factor 2 and 2 grids with second highest loadings but surrounded by grids with the highest loadings. However, this square includes 2 grids (27.5 °N 112.5 °E, 27.5 °N 114.5 °E) without neither the highest nor second highest loadings over SEC, which were excluded for this pattern.

4 Eastern Tibetan Plateau (ETP) pattern covers a square area of 29.5-35.5°N 94.5-104.5°E and a neighboring area with three grids from92.5-96.5°E at 27.5°N. This pattern includes 12 grids with the highest loadings over factor 6 and 3 grids surrounded by these grids showing second highest loading on factor 6. There are 4 grids within this area but excluded in this pattern as they have neither the highest nor second highest loadings over the ETP pattern.

5. The lower reaches of the Yangtze river (LYR) pattern includes a square area of 29.5-31.5°N 110.5-120.5 °E and 4 nearby grids at 27.5 °N 114.5 °E, 33.5 °N 110.5 °E, 33.5 °N 112.5 °E and 35.5 °N 112.5 °E. The LYR pattern includes 11 grids with the highest loadings over factor 4 and 5 grids with the second highest loadings but by the grids with the highest loadings.

6. The north-central China (NCC) pattern includes a square area of 35.5-39.5 °N 108.5-114.5 °E. This pattern includes 6 grids with the highest loadings over factor 6 and 2 grids with the second highest loading and surrounded by the grids with the highest loadings. One grid within this square area but without neither the highest nor the second heighest loadings were excluded in this pattern.

7. The Bohai Sea Rim (BSR) includes a square area of 35.5-41.5 °N 118.5-122.5 °E. This pattern includes 9 grids with the highest loadings over factor 5 and 1 grids with the second highest loadings and surrounded by the grids with the highest loadings. There is one grid (37.5 °N 118.5 °E) surrounded by the grids of the highest loadings but excluded in this pattern as it has neither the highest nor second highest loadings over factor 5.

8. The Northwestern China (NWC) pattern includes an area of 43.5-47.5°N 80.5-91.5°E. This pattern includes 6 grids with the highest loadings over factor 1 and 1 grids surrounded by these grids and having the second highest loading. There are 5 grids (45.5°N 82.5°E, 47.5°N 82.5°E, 43.5°N 84.5°E, 47.5°N 84.5°E and 43.5°N 86.5°E) in this area but without neither the highest nor the second highest loadings over factor 6, which are not included in this pattern.

9. Northeastern China was divided into western and eastern parts (WNE and ENE). WNE includes ten neighboring grids within an area of 43.5-51.5 °N 122.5-128.5 °E. This pattern includes 8 grids with the highest loadings over factor 2 and 2 grids showing the second highest loadings and surrounded by these grids with the highest loadings.

10. The eastern part of northeastern China (ENE) includes a square area of 47.5-49.5°N 126.5-130.5°E. This pattern includes 3 grids with the highest loadings over factor 11 and 2 grids within this area, showing the second highest loading. There is a grid (49.5°N 126.5°E) excluded in this pattern as it has neither the highest nor second highest loadings on factor 11.


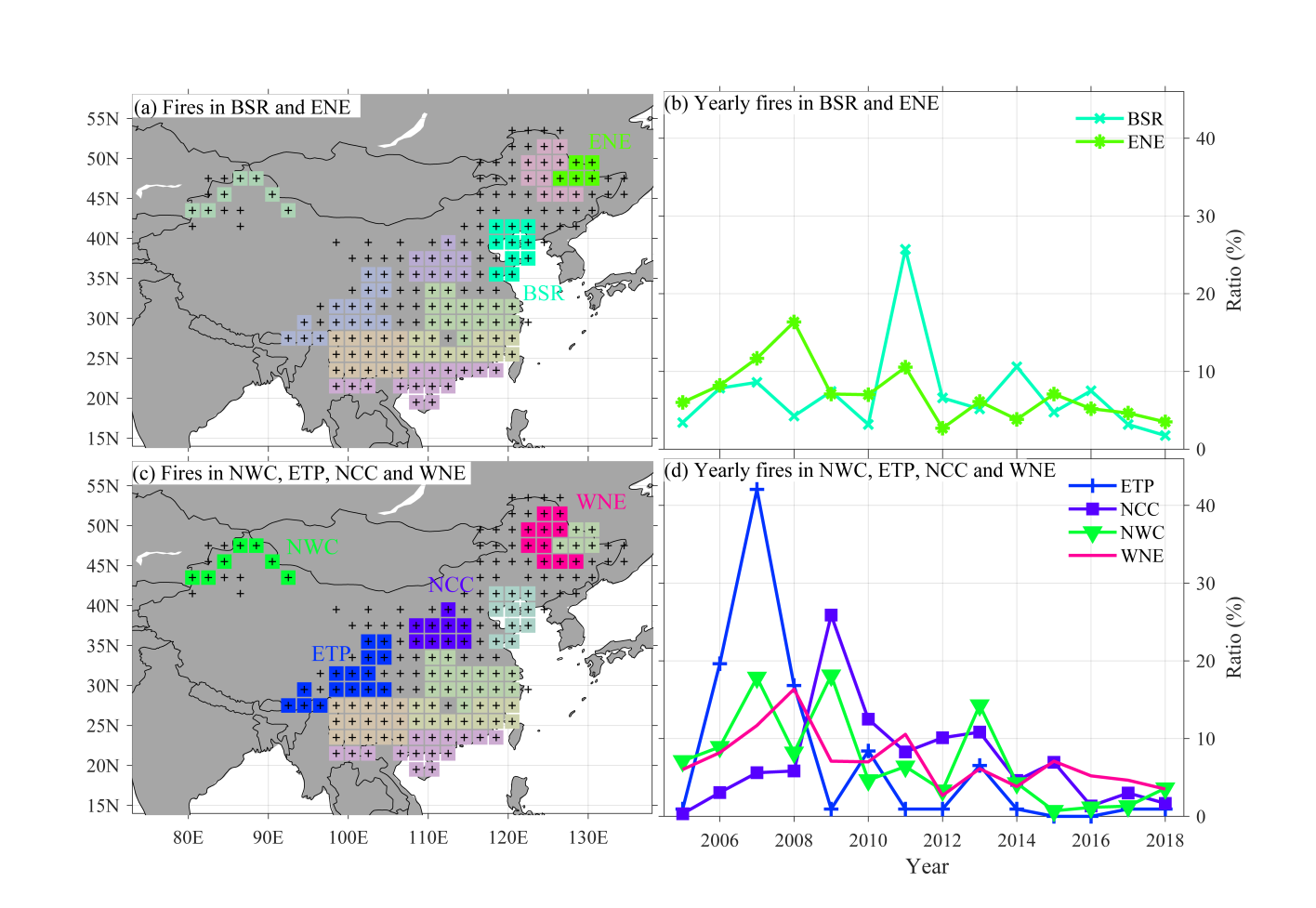


**Supplementary Figure 1**. The (a) fire regions of the Bohai Sea Rim (BSR) and the eastern part of northeastern China (ENE), (b) ratio (percentage between fire of a year and all years) of fire chronologies of the BSR and ENE, the (c) fire regions of northwestern China (NWC), eastern Tibetan Plateau (ETP), north-central China (NCC) and the western part of northeastern China (WNE), and (d) the ratio of fire chronologies of the NWC, ETP, NCC and WNE. The six groups are shown separately in two groups (BSR and ENE) and the other four (NWC, ETP, NCC and WNE) according to their correlations. The other shaded areas are the other fire regions shown in the main text.


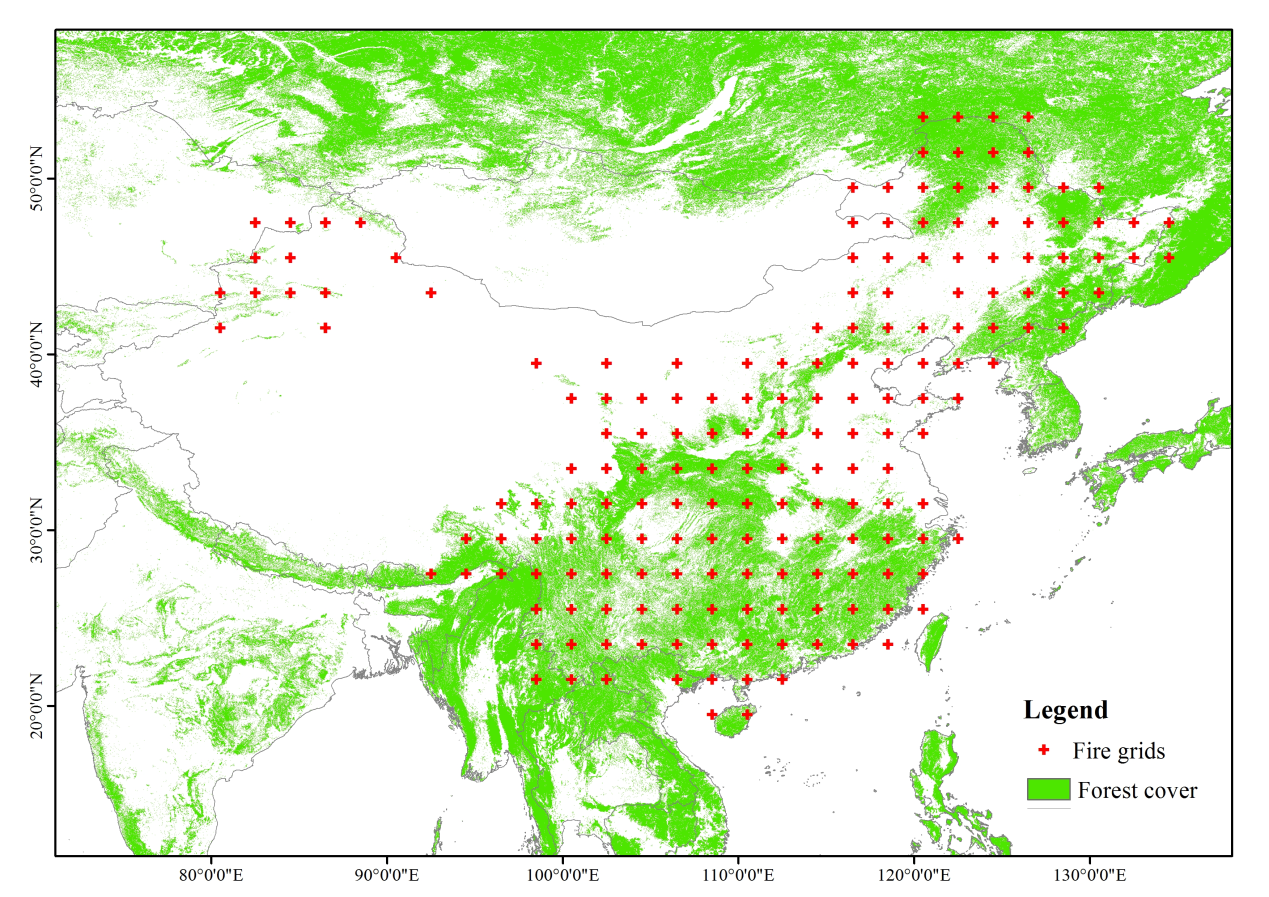


**Supplementary Figure 2**. Map showing the grid locations of the Wildfire Atlas of China (WFAC) and the forest cover. The forest covers were derived from the earth land cover map ^2^.


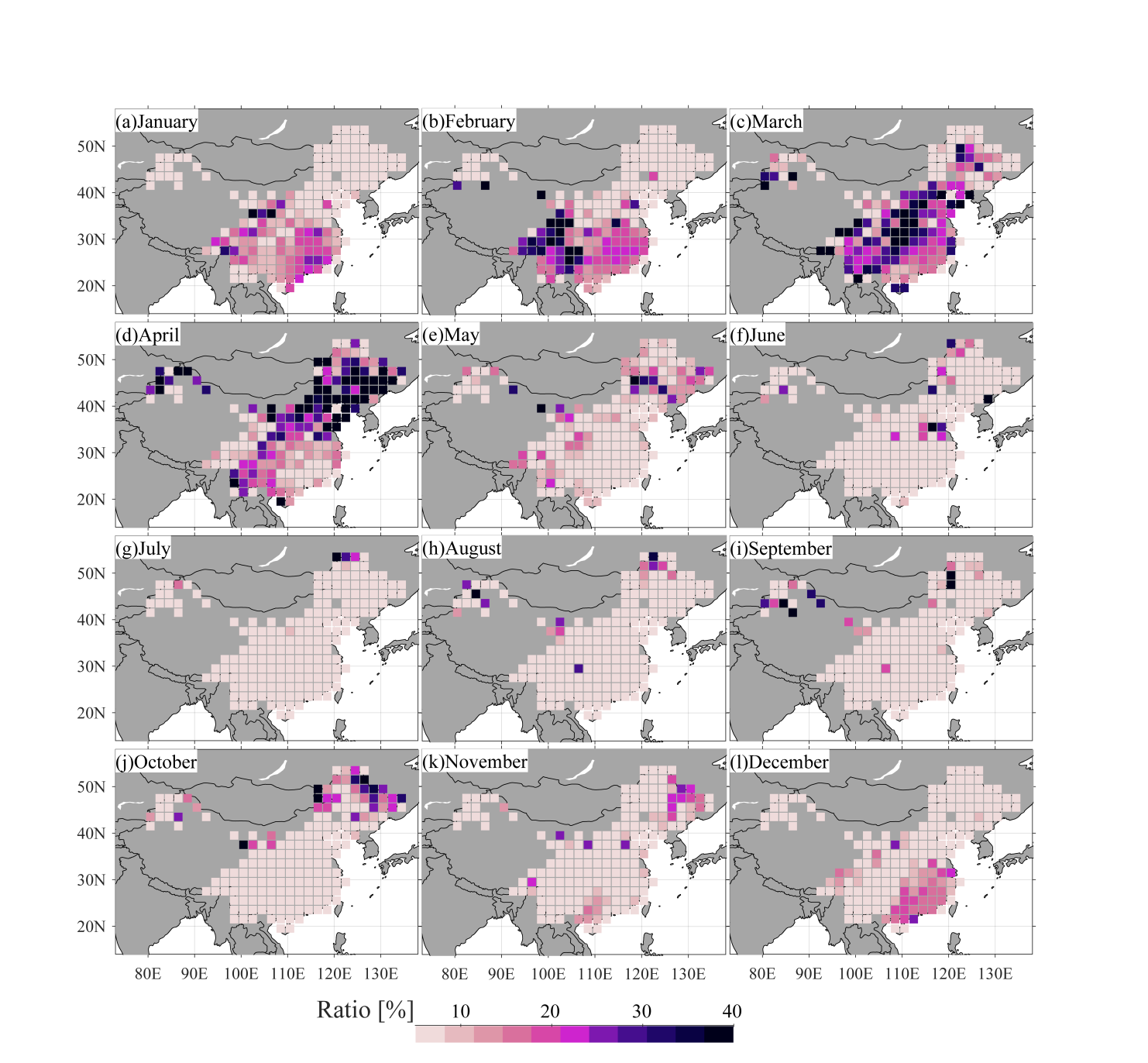


**Supplementary Figure 3**. Monthly distribution of the percent of the fire numbers from 2005-2018. The percentages were calculated as the ratio between monthly mean fires numbers and the total fire numbers.


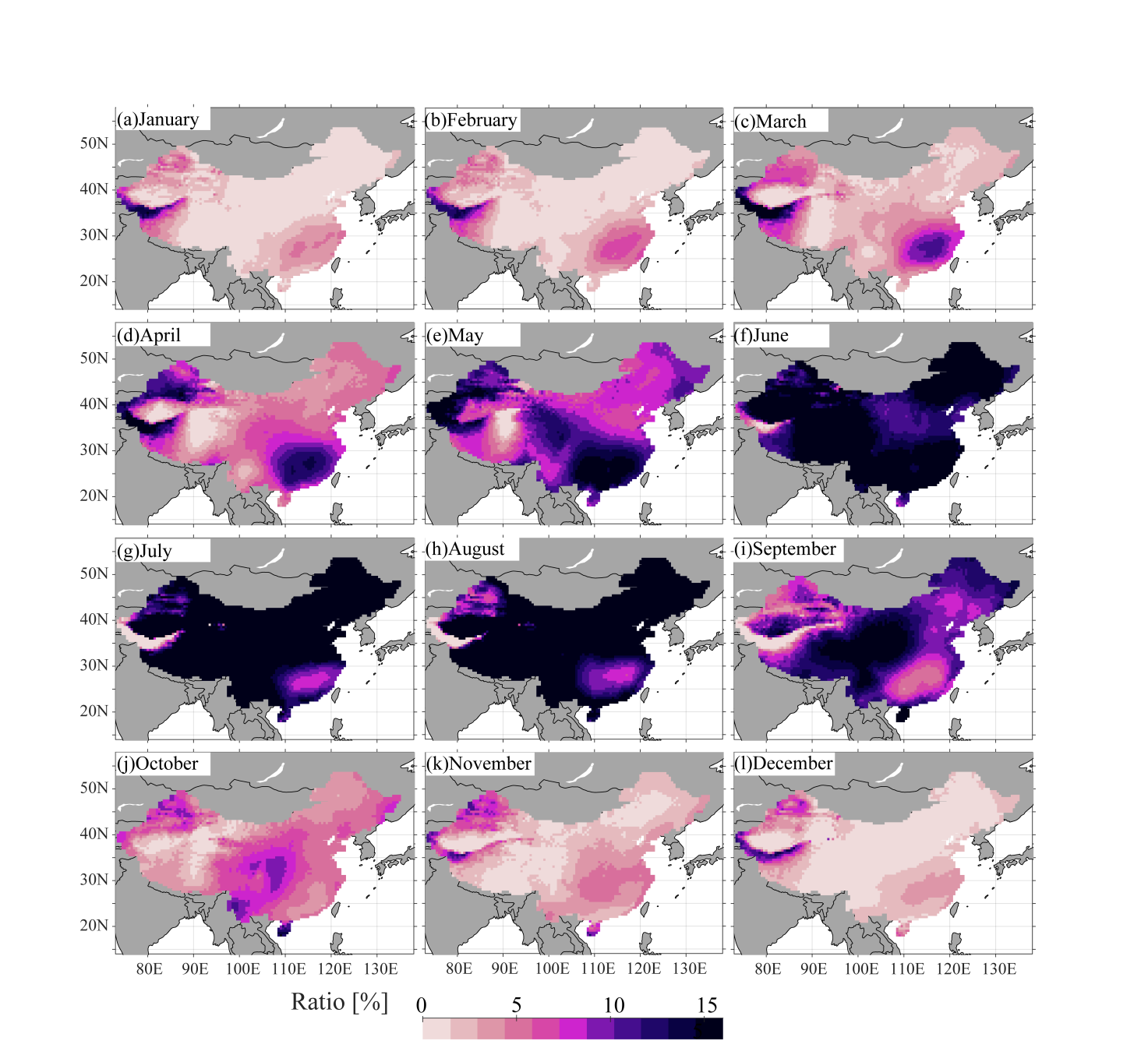


**Supplementary Figure 4**. Monthly distribution of the percent of the precipitation in mainland China based on the Climate Research Unit dataset (CRU TS4.03) data. The monthly precipitation percents were calculated as the ratio between monthly mean precipitation and the yearly total precipitation spanning from 1950 to 2018 when most meteorological stations were developed.


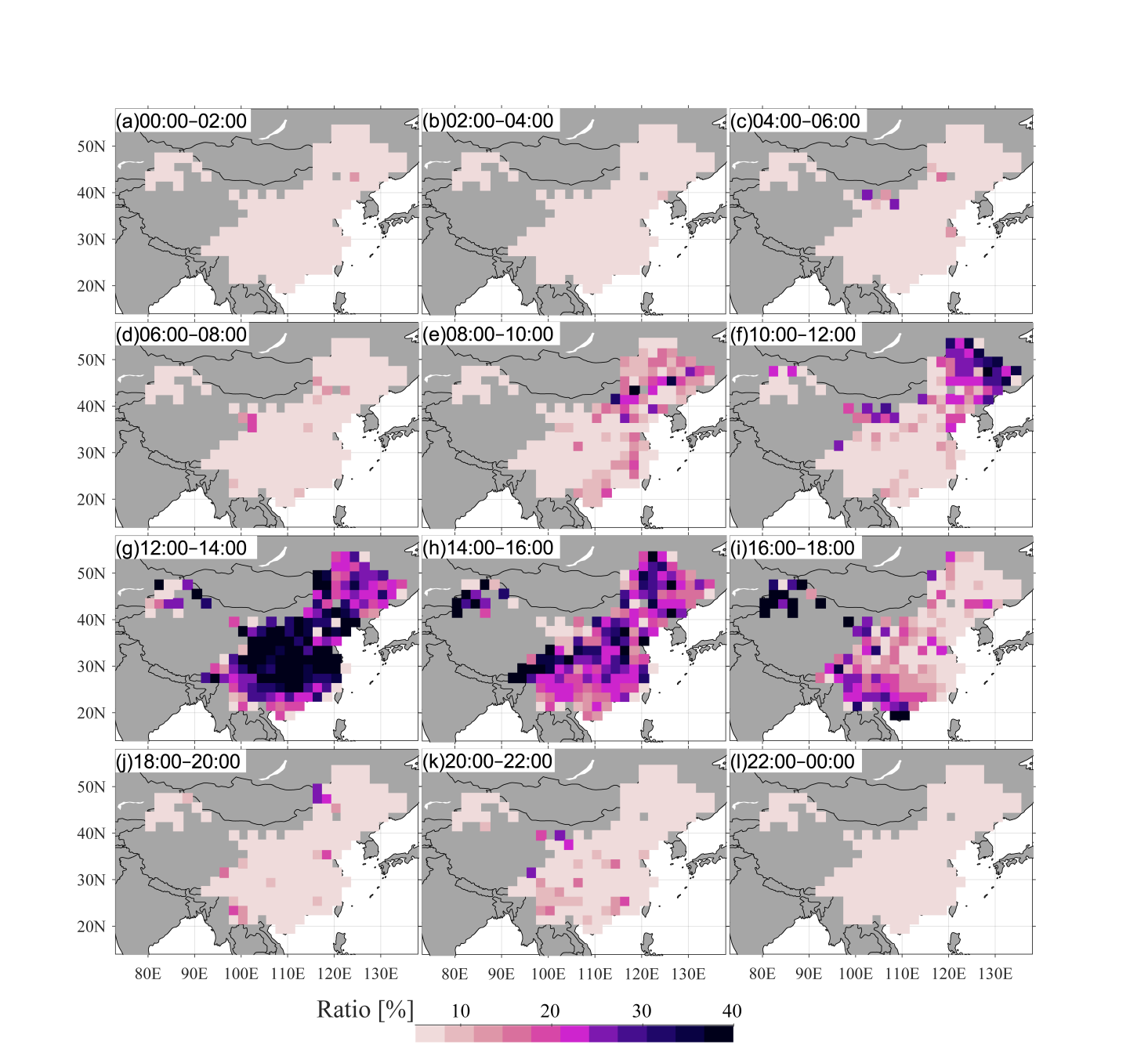


**Supplementary Figure 5**. Hourly distribution of the percent of the fire numbers from 2005-2018 at a step of 2 hours. The percentages were calculated as the ratio between hourly mean fires numbers and the total fire numbers.


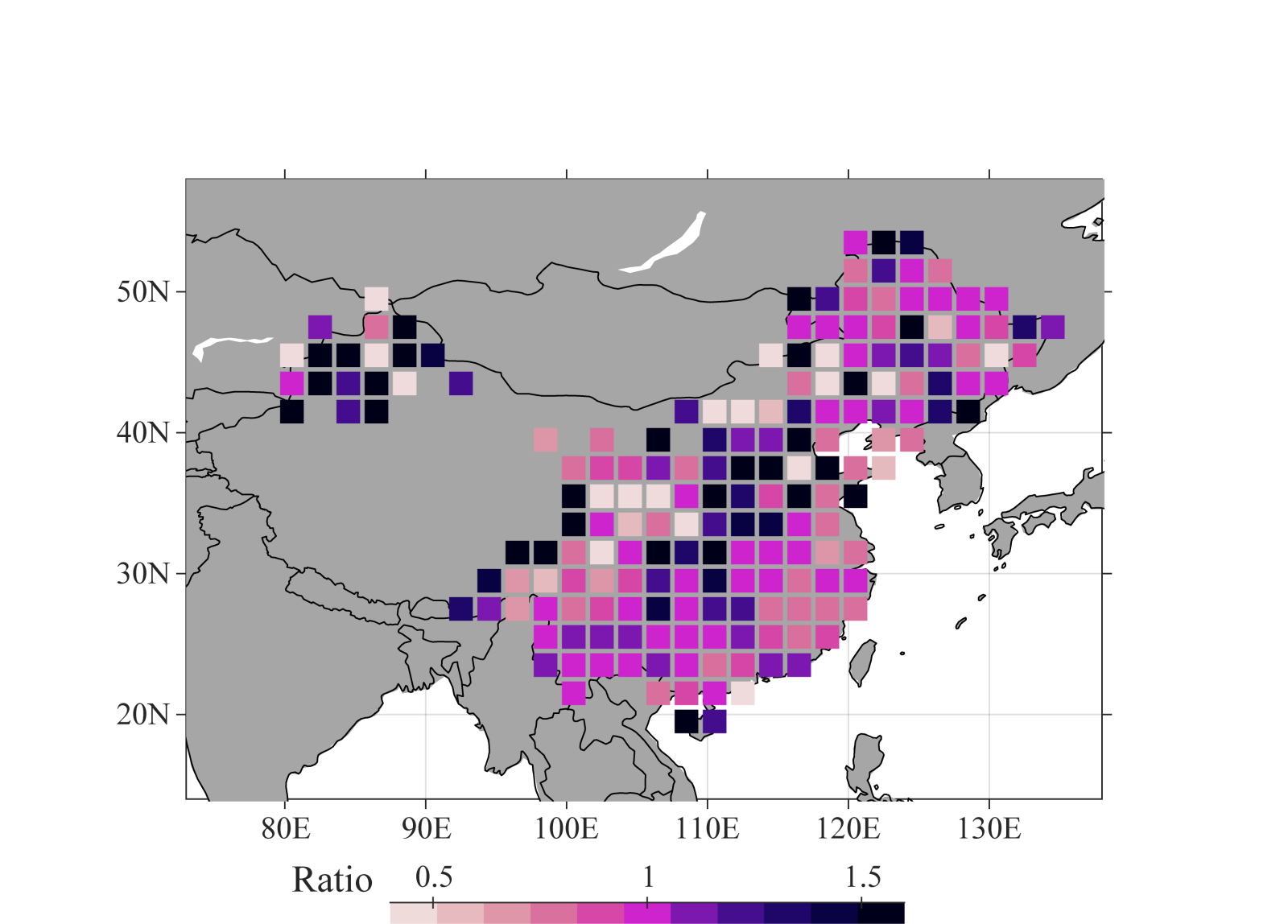


**Supplementary Figure 6**. Map of the ratio between the daily mean fire number of the weekend (Saturday and Sunday) and weekday (Monday to Friday).


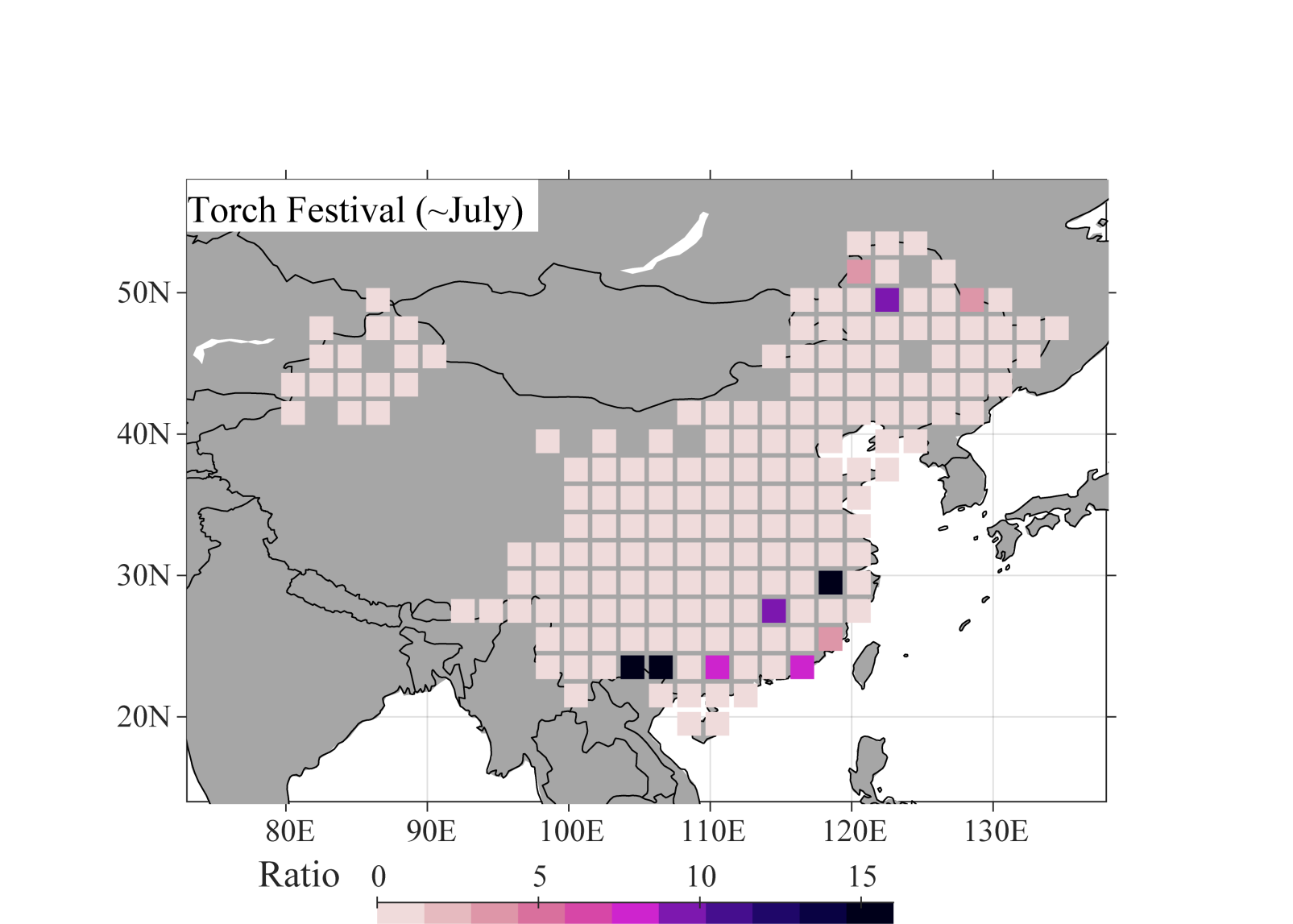


**Supplementary Figure 7**. Maps of the ratio between the fire numbers of the Torch Festival and the daily mean number of fires in the month with Torch Festival. The Torch Festival is a traditional festival especially for the Yi minority and some other minorities in southeastern China. People celebrate this festival by blazing torches on this festival. The celebration occurs on June 24^th^ according to the traditional Chinese Lunar Calendar, which vary in date in different years (July 29^th^ of 2005, July 19^th^ of 2006, August 6^th^ of 2007, July 26^th^ of 2008, August 14^th^ of 2009, August 4^th^ of 2010, July 24^th^ of 2011, August 11^th^ of 2012, July 31^st^ of 2013, July 20^th^ of 2014, August 8^th^ of 2016, July 27^th^ of 2016, July 17^th^ of 2017 and August 5^th^ of 2018).


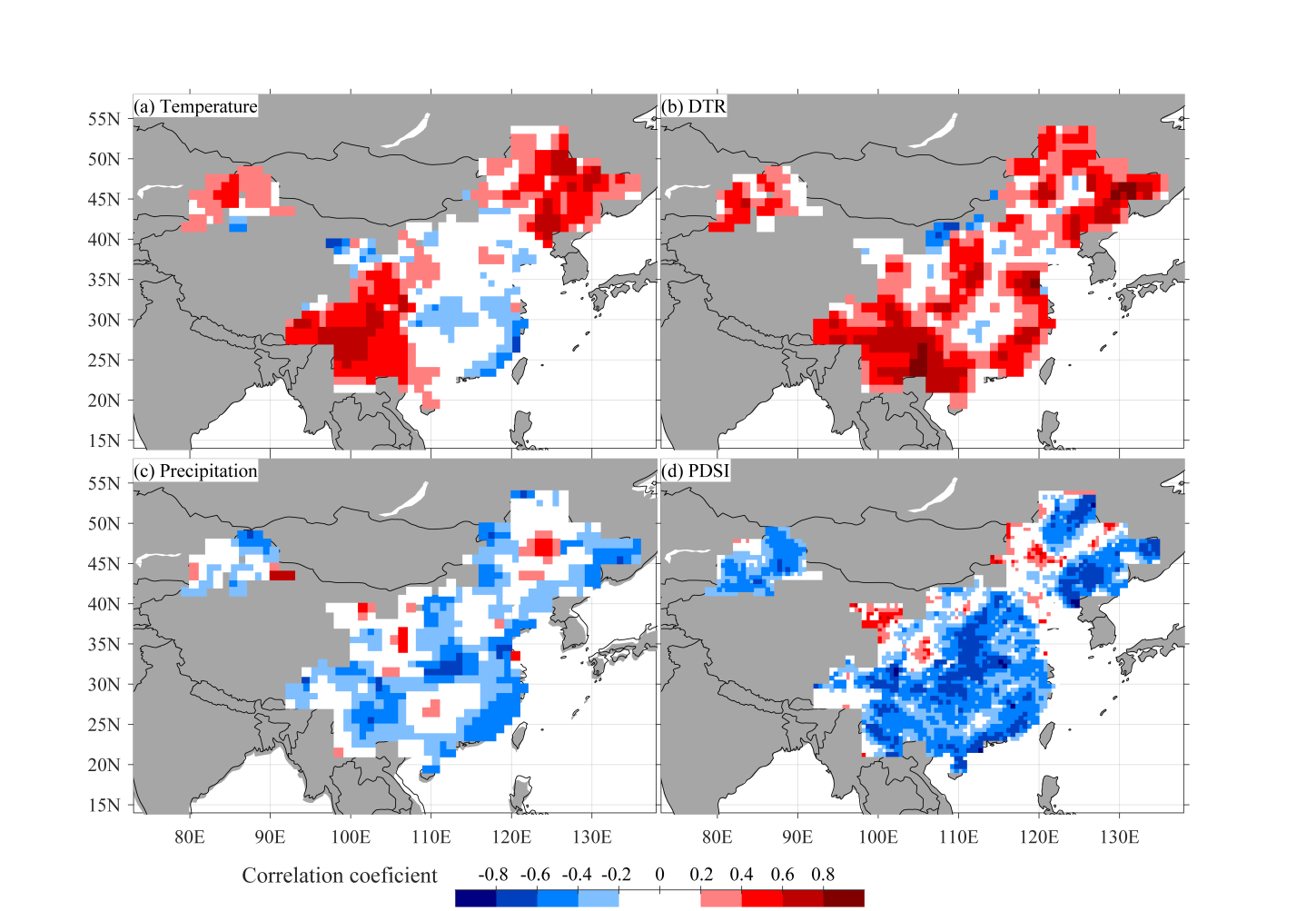


**Supplementary Figure 8**. Pointwise correlations between the unfiltered (not being first differenced) number of WFAC gridded fires and the (a) temperature, (b) diurnal temperature range (DTR), (c) precipitation and (d) Palmer drought severity index (PDSI) in the main fire season from January to April.

**
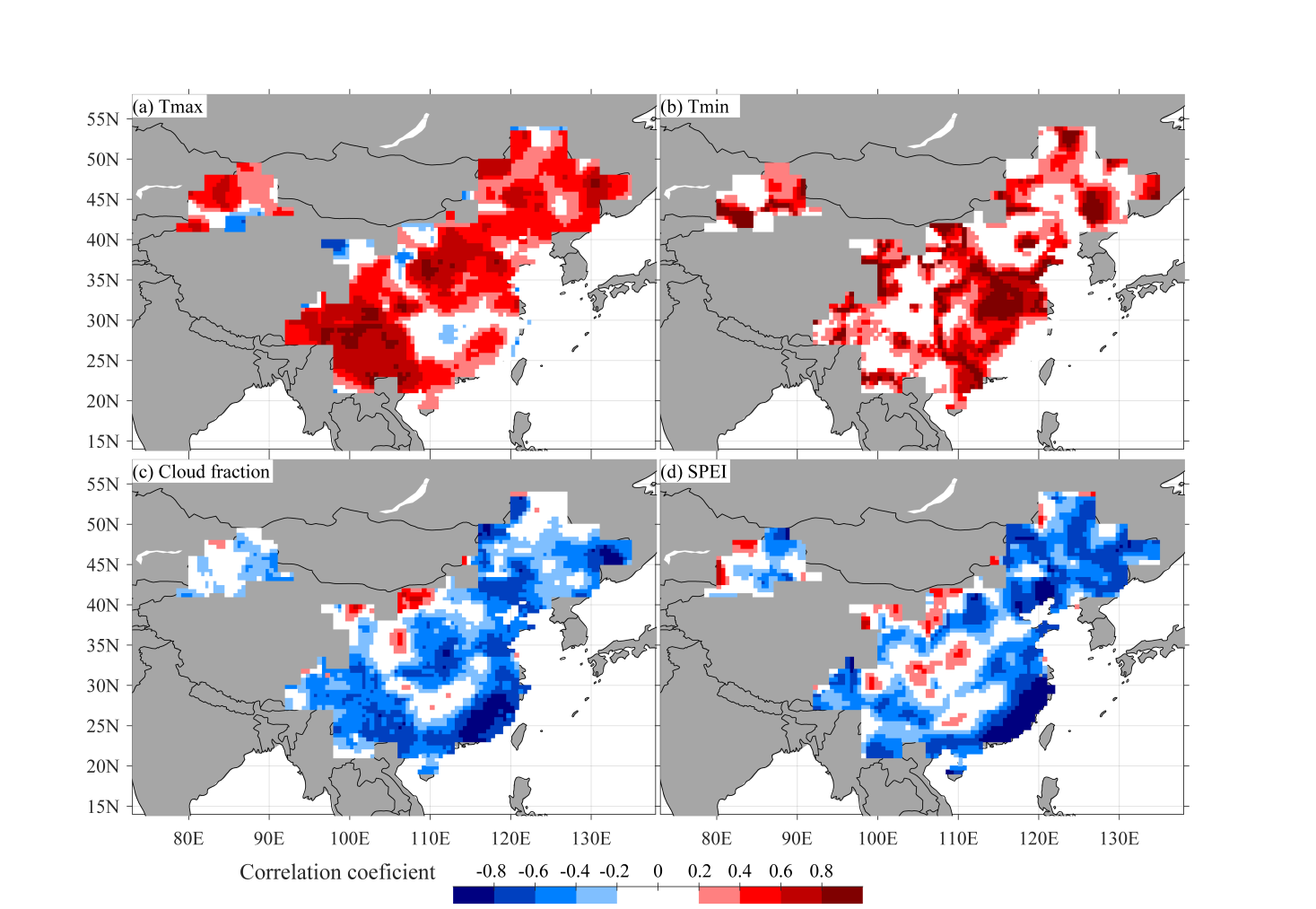
**

**Supplementary Figure 9**. Pointwise correlations between the first differenced number of gridded fires and the (a) maximum temperature (Tmax), (b) minimum temperature (Tmin), (c) cloud fraction and (d) Standardised Precipitation-Evapotranspiration Index (SPEI) in the main fire season from January to April. The maximum and minimum temperatures and cloud fraction data were derived into the 0.5°×0.5° gridded Climate Research Unit dataset (CRU TS4.03) data spanning from 1901 to 2018 ^3^. The SPEI is a multiple-timescale drought index ^4^ and were calculated from the CRU data with a 0.5°× 0.5° spatial resolution. The first differenced data are the residuals between data of two successive years.


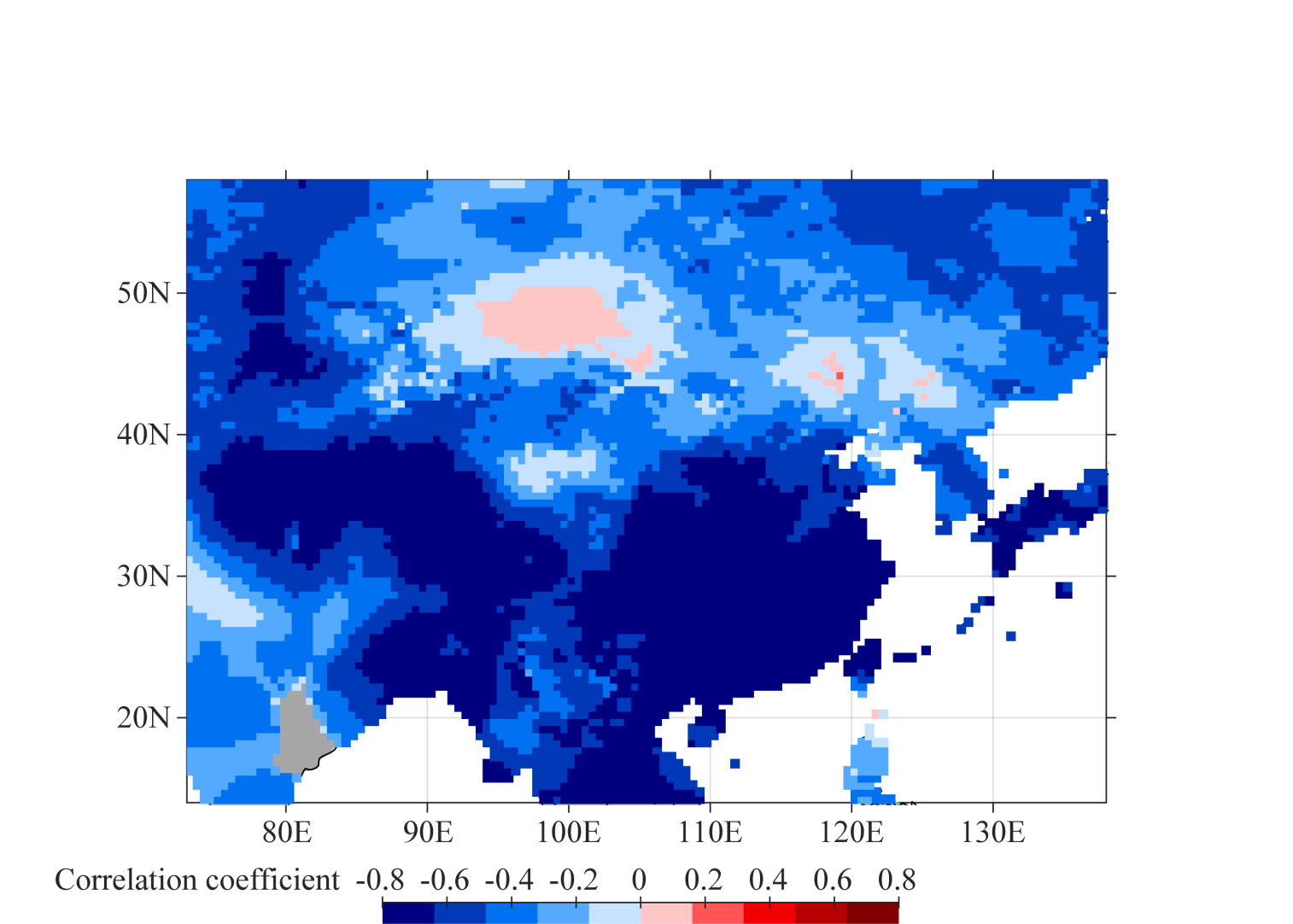


**Supplementary Figure 10**. Map of correlations between the diurnal temperature range (DTR) and cloud fraction during the fire season from January to April from 1950 to 2019. The DTR data are the same as in Fig. 4 and the cloud fraction data are the same as in Figure 4. As shown in the correlation map, the negative correlations between DTR and cloud fractions are significant over most of southern China, where most of the fires occurred.


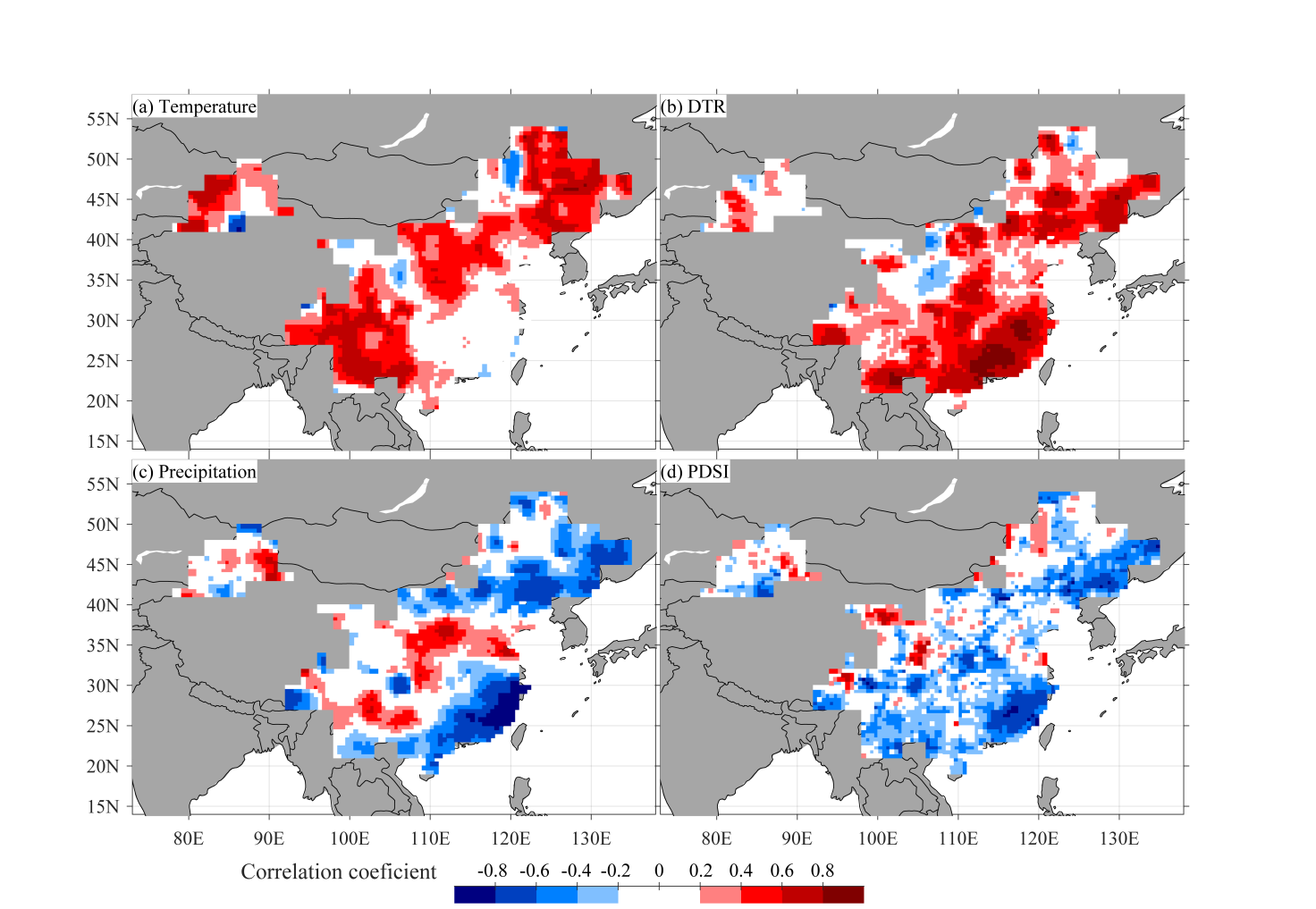


**Supplementary Figure 11**. Pointwise correlations between the gridded first differenced fire number and the (a) temperature, (b) diurnal temperature range (DTR), (c) precipitation and (d) Palmer Drought Severity Index (PDSI) for annual data from January to December. The data used are the same as Fig. 4 in the main text. The first differenced data are the residuals between data of two successive years.


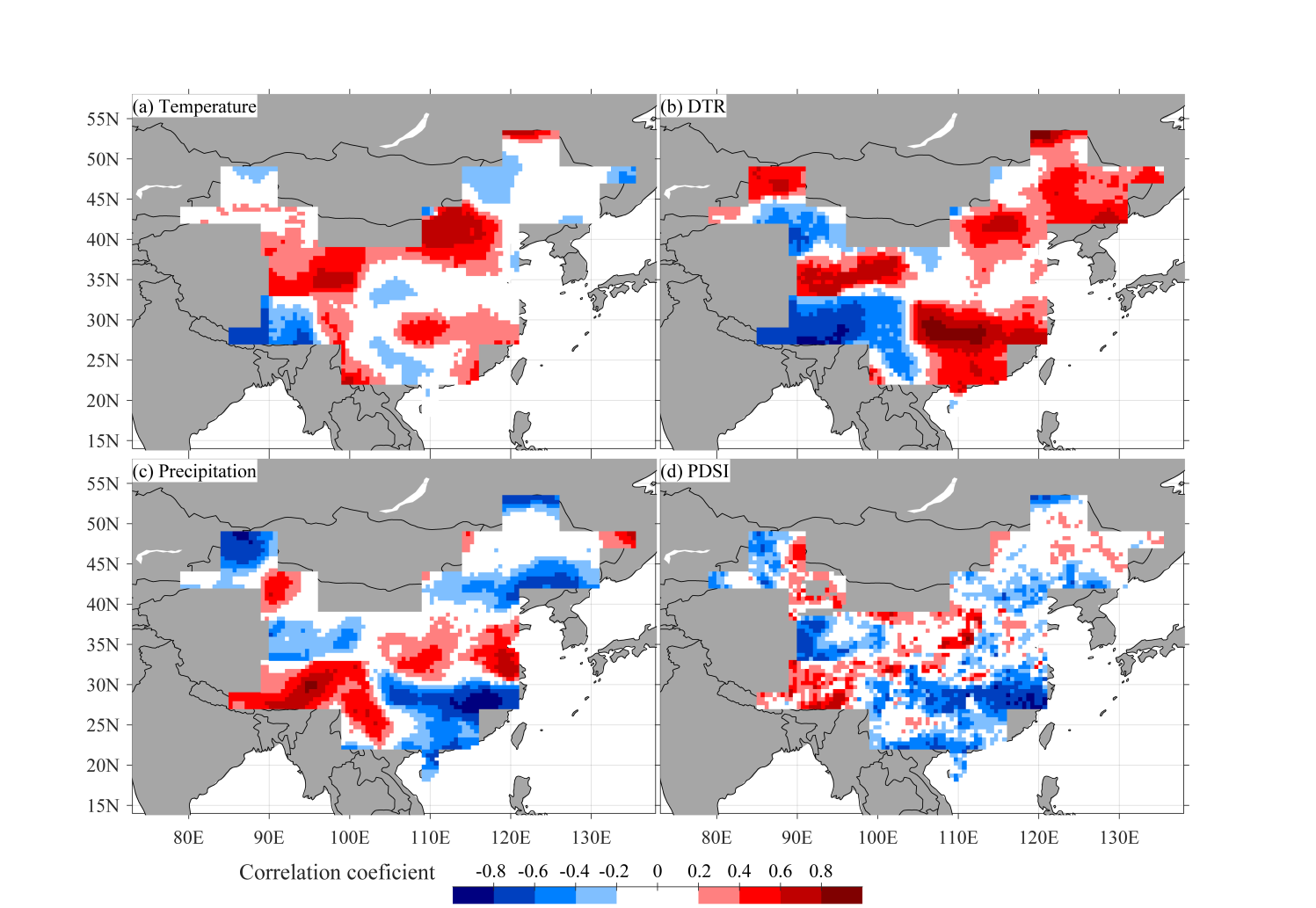


**Supplementary Figure 12**. Pointwise correlations between the first differenced fire numbers (2005-2018) and average (a) temperature, (b) diurnal temperature range (DTR), (c) precipitation, and (d) Palmer drought severity index (PDSI) during the monsoon season from May to September. The first differenced data are the residuals between data of two successive years.


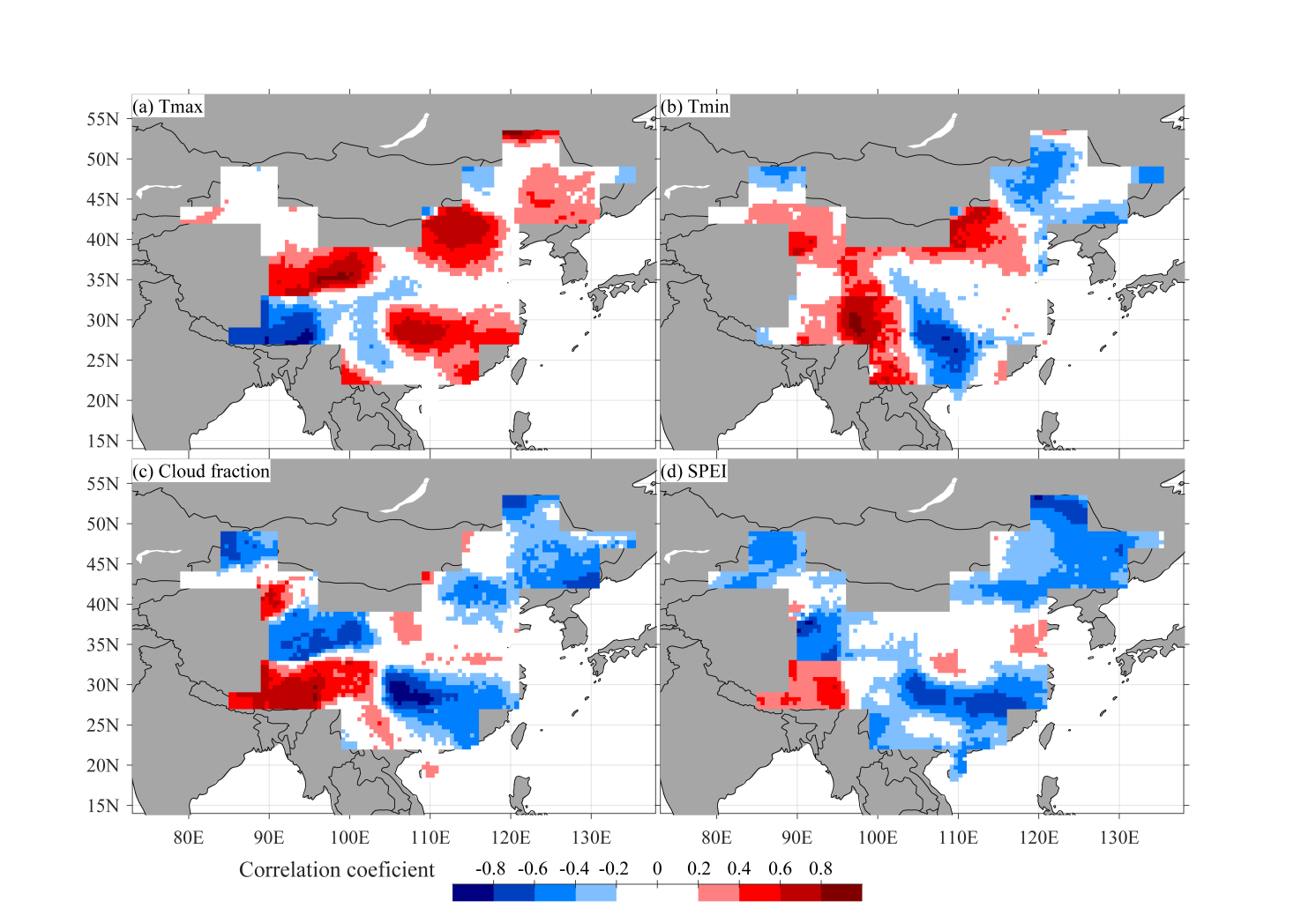


**Supplementary Figure 13**. Pointwise correlations between the first differenced number of gridded fires and the (a) maximum temperature (Tmax), (b) minimum temperature (Tmin), (c) cloud fraction and (d) Standardised Precipitation-Evapotranspiration Index (SPEI) in the monsoon season from May to September. The maximum and minimum temperatures and cloud fraction data were derived into the 0.5°×0.5° gridded Climate Research Unit dataset (CRU TS4.03) data spanning from 1901 to 2018 ^3^. The SPEI is a multiple-timescale drought index ^4^ and were calculated from the CRU data with a 0.5°× 0.5° spatial resolution. The first differenced data are the residuals between data of two successive years.


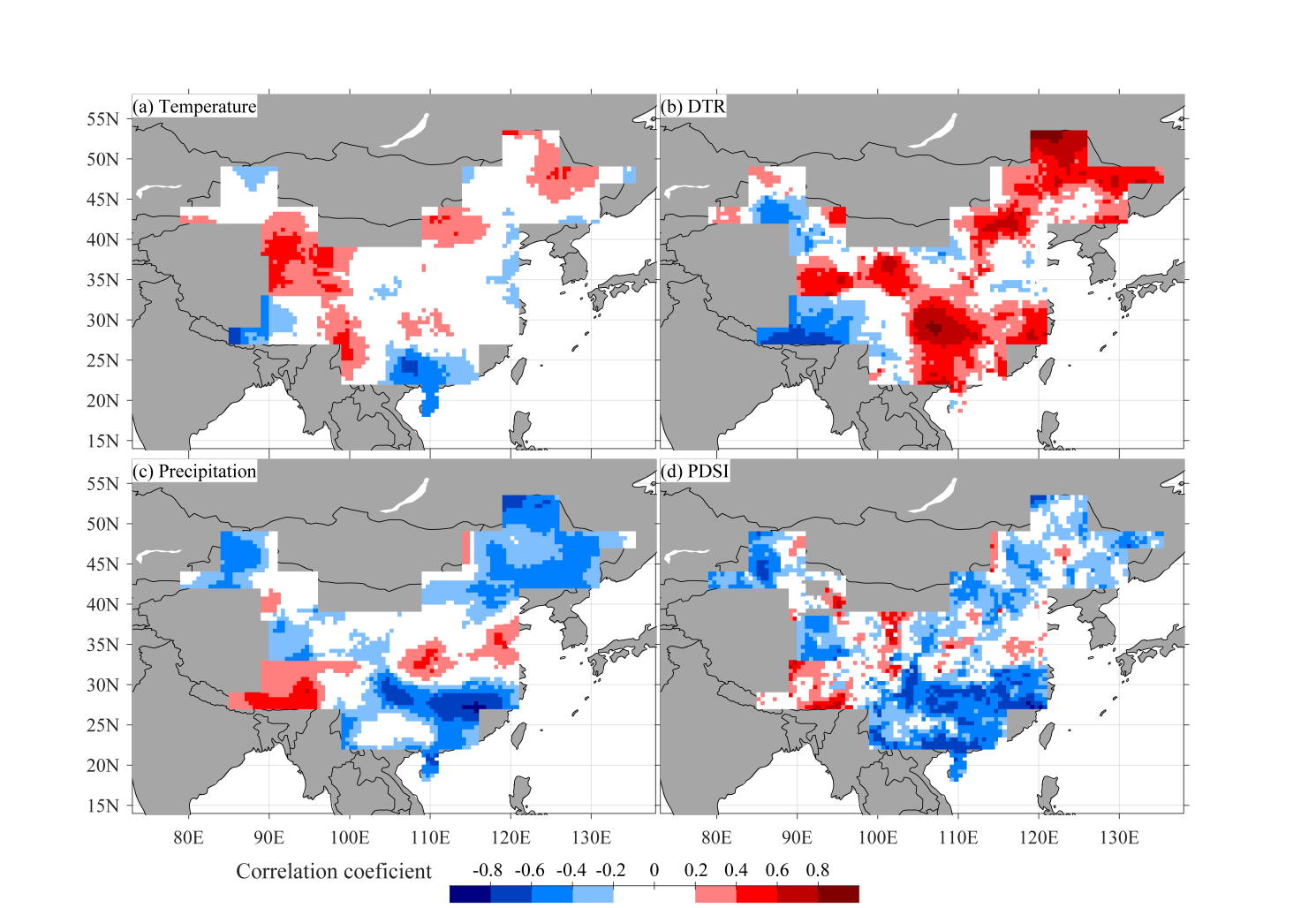


**Supplementary Figure 14**. Pointwise correlations between the unfiltered (not being first differenced) fire numbers (2005-2018) and average (a) temperature, (b) diurnal temperature range (DTR), (c) precipitation, and (d) Palmer drought severity index (PDSI) during the monsoon season from May to September.


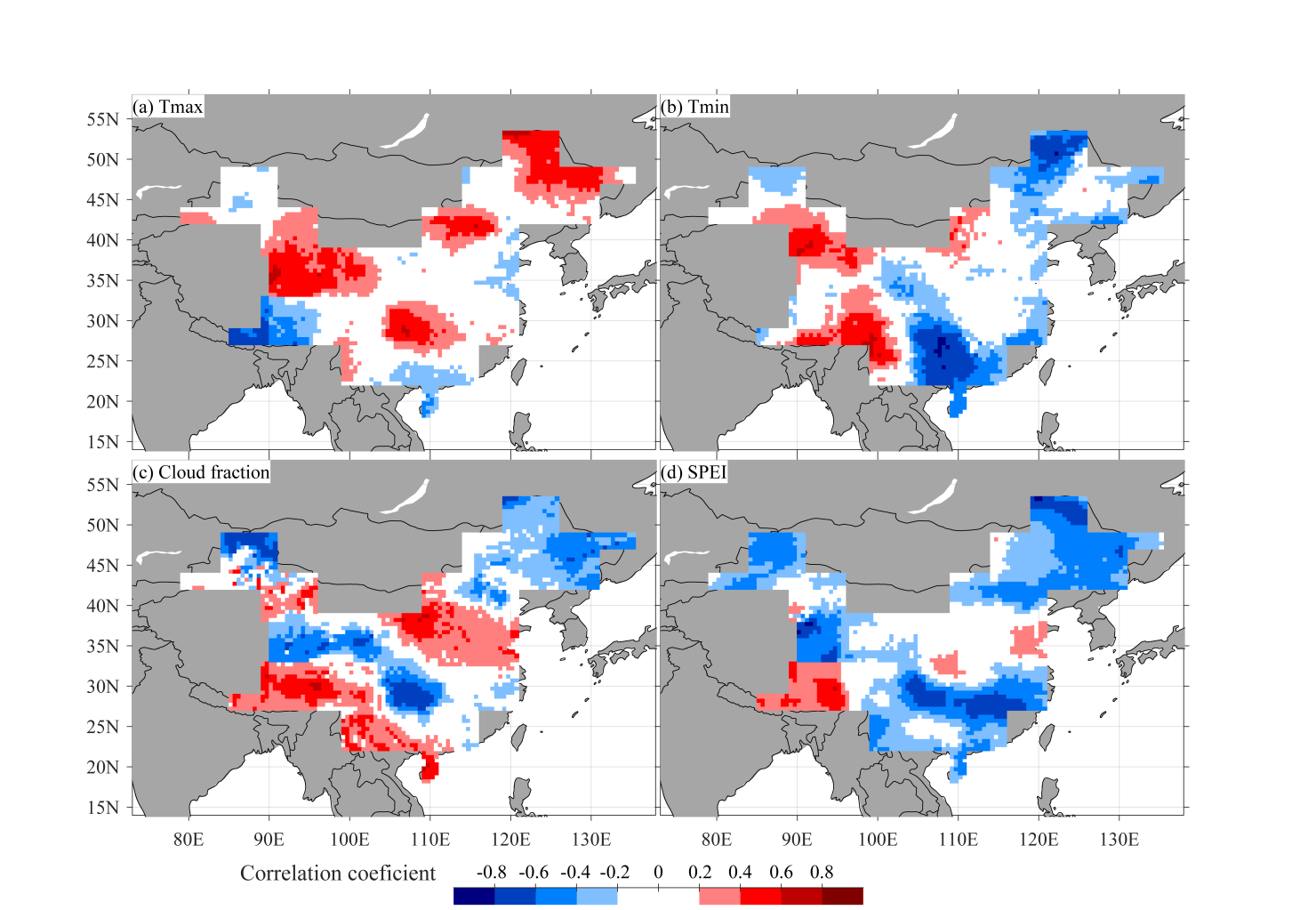


**Supplementary Figure 15**. Pointwise correlations between the unfiltered (not being first differenced) fire numbers and the (a) maximum temperature (Tmax), (b) minimum temperature (Tmin), (c) cloud fraction and (d) Standardised Precipitation-Evapotranspiration Index (SPEI) in the monsoon season from May to September. The maximum and minimum temperatures and cloud fraction data were derived into the 0.5°×0.5° gridded Climate Research Unit dataset (CRU TS4.03) data spanning from 1901 to 2018 ^3^. The SPEI is a multiple-timescale drought index ^4^ and were calculated from the CRU data with a 0.5°× 0.5° spatial resolution.


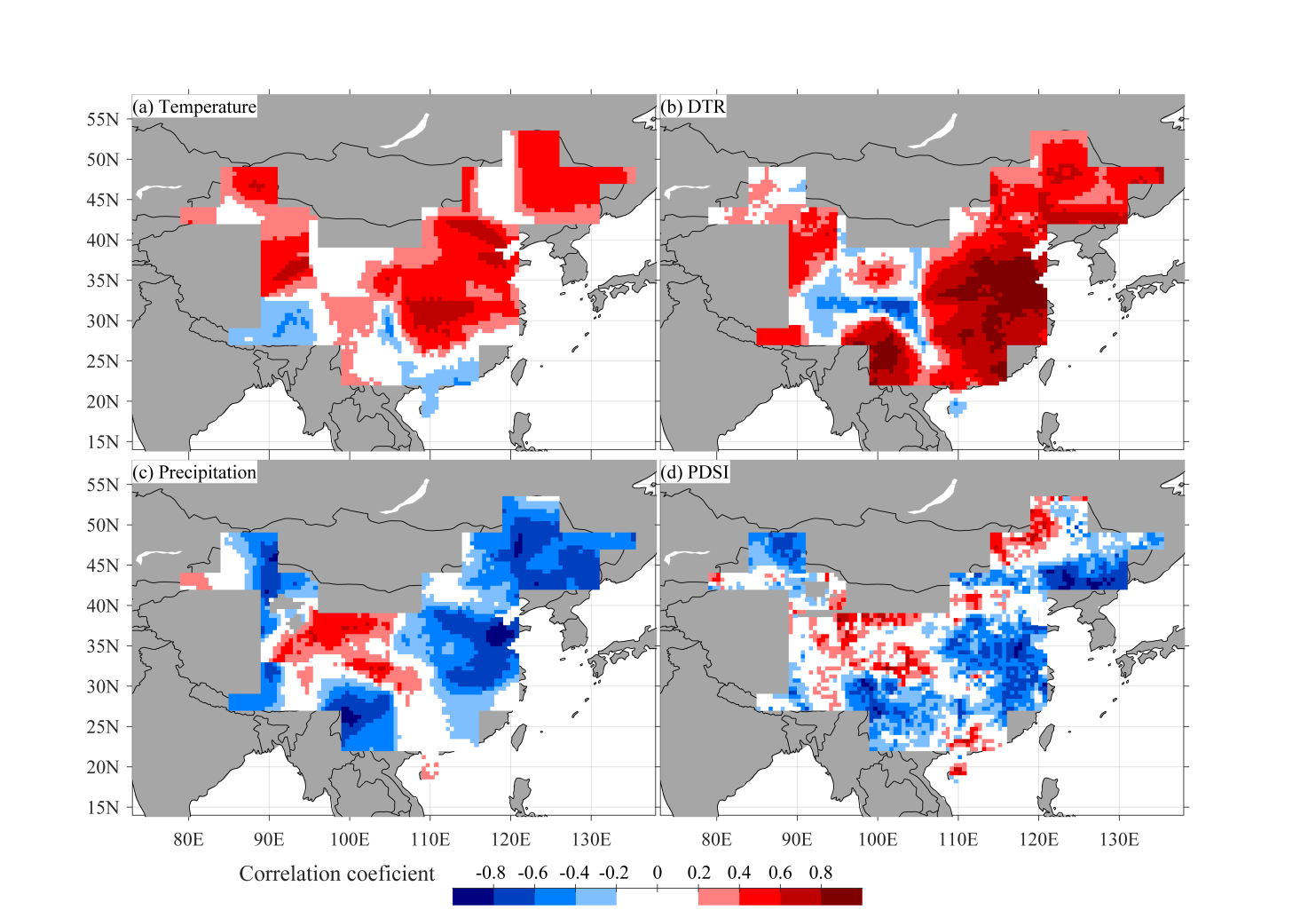


**Supplementary Figure 16**. Pointwise correlations between the first differenced fire numbers (2005-2018) and average (a) temperature, (b) diurnal temperature range (DTR), (c) precipitation, and (d) Palmer drought severity index (PDSI) during the post-monsoon season from October to December. The first differenced data are the residuals between data of two successive years.


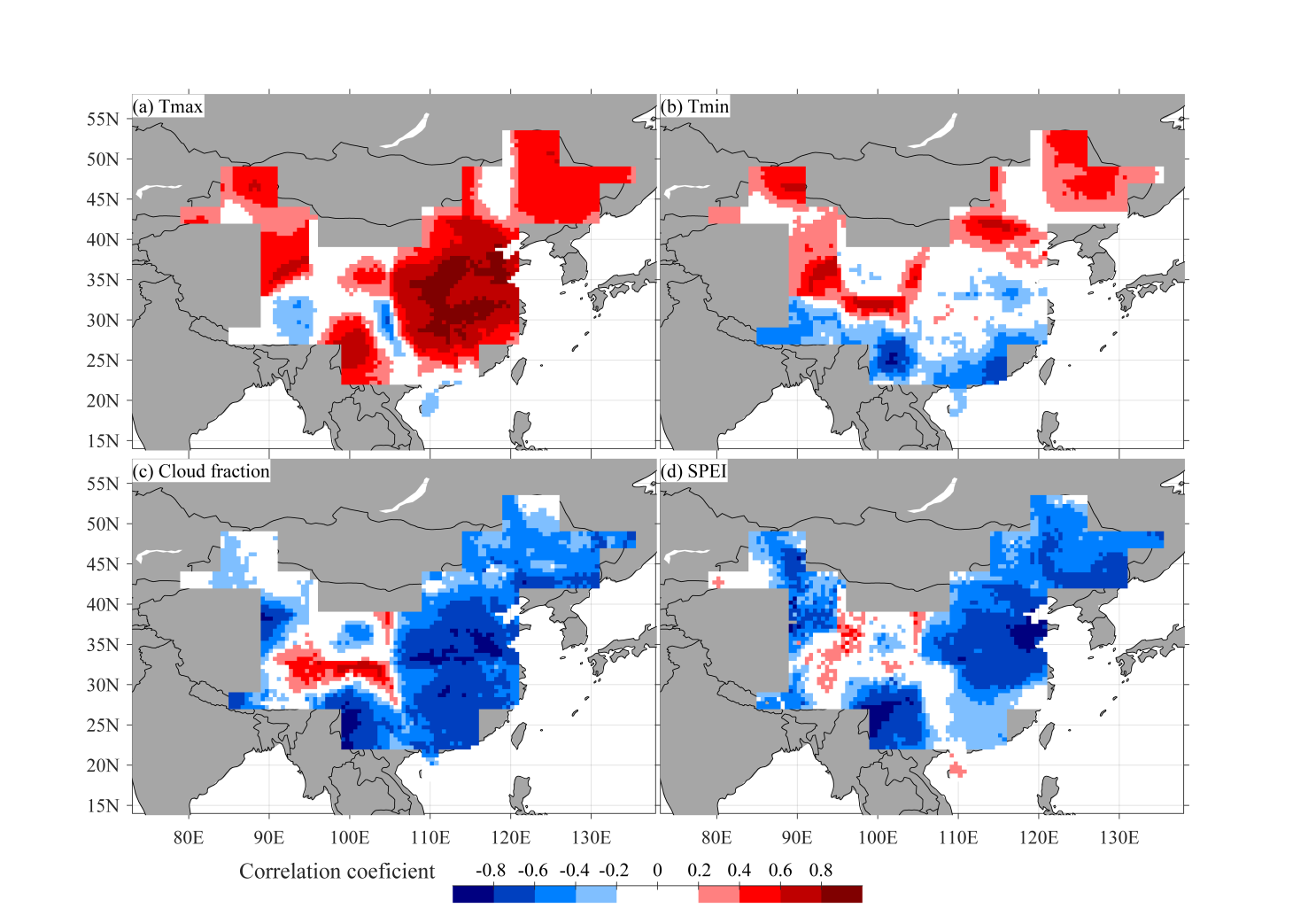


**Supplementary Figure 17**. Pointwise correlations between the first differenced number of gridded fires and the (a) maximum temperature (Tmax), (b) minimum temperature (Tmin), (c) cloud fraction and (d) Standardised Precipitation-Evapotranspiration Index (SPEI) in the post-monsoon season from October to December. The maximum and minimum temperatures and cloud fraction data were derived into the 0.5°×0.5° gridded Climate Research Unit dataset (CRU TS4.03) data spanning from 1901 to 2018 ^3^. The SPEI is a multiple-timescale drought index ^4^ and were calculated from the CRU data with a 0.5°× 0.5° spatial resolution. The first differenced data are the residuals between data of two successive years.


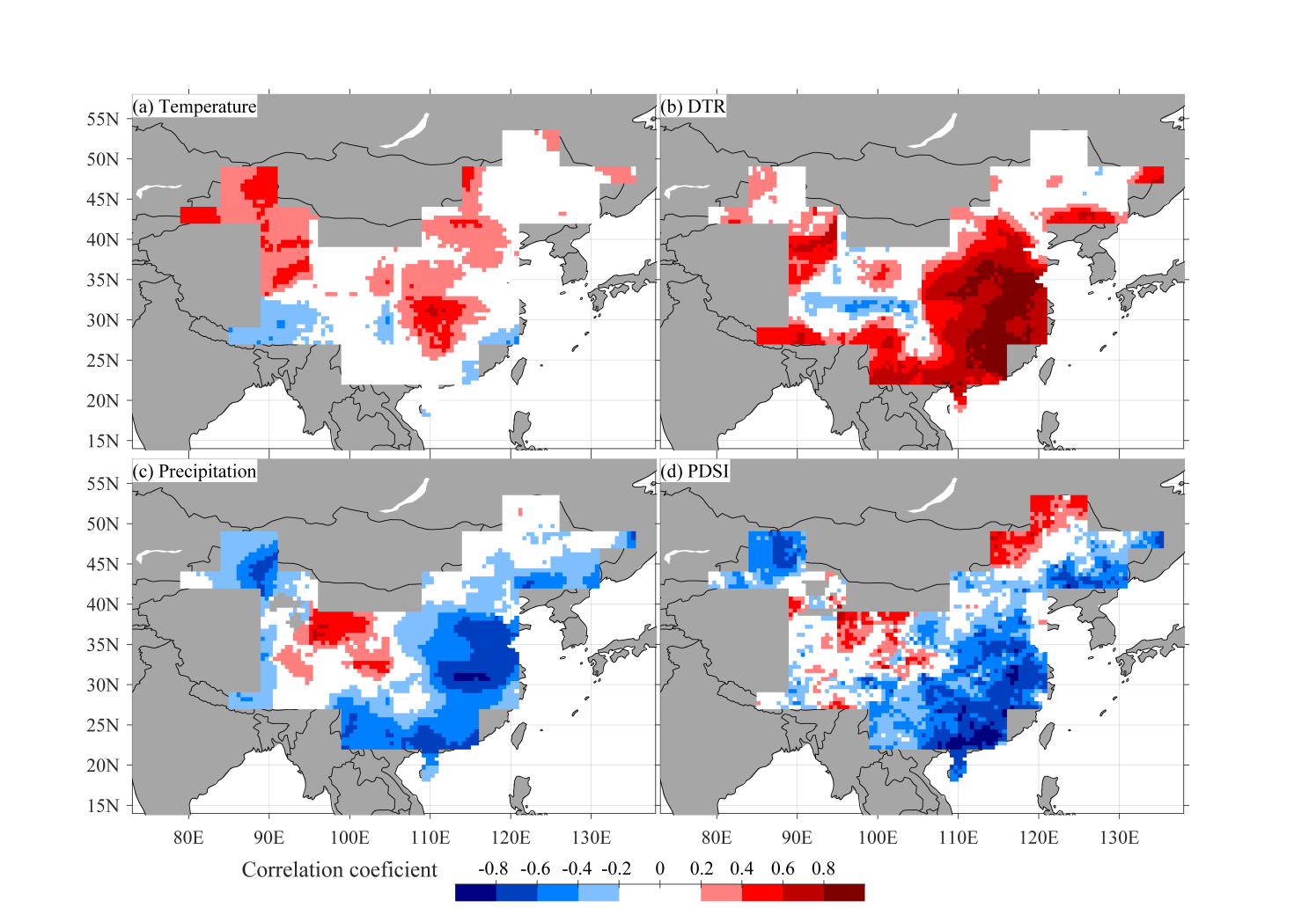


**Supplementary Figure 18**. Pointwise correlations between the unfiltered (not being first differenced) fire numbers (2005-2018) and average (a) temperature, (b) diurnal temperature range (DTR), (c) precipitation, and (d) Palmer drought severity index (PDSI) during the post-monsoon season from October to December.


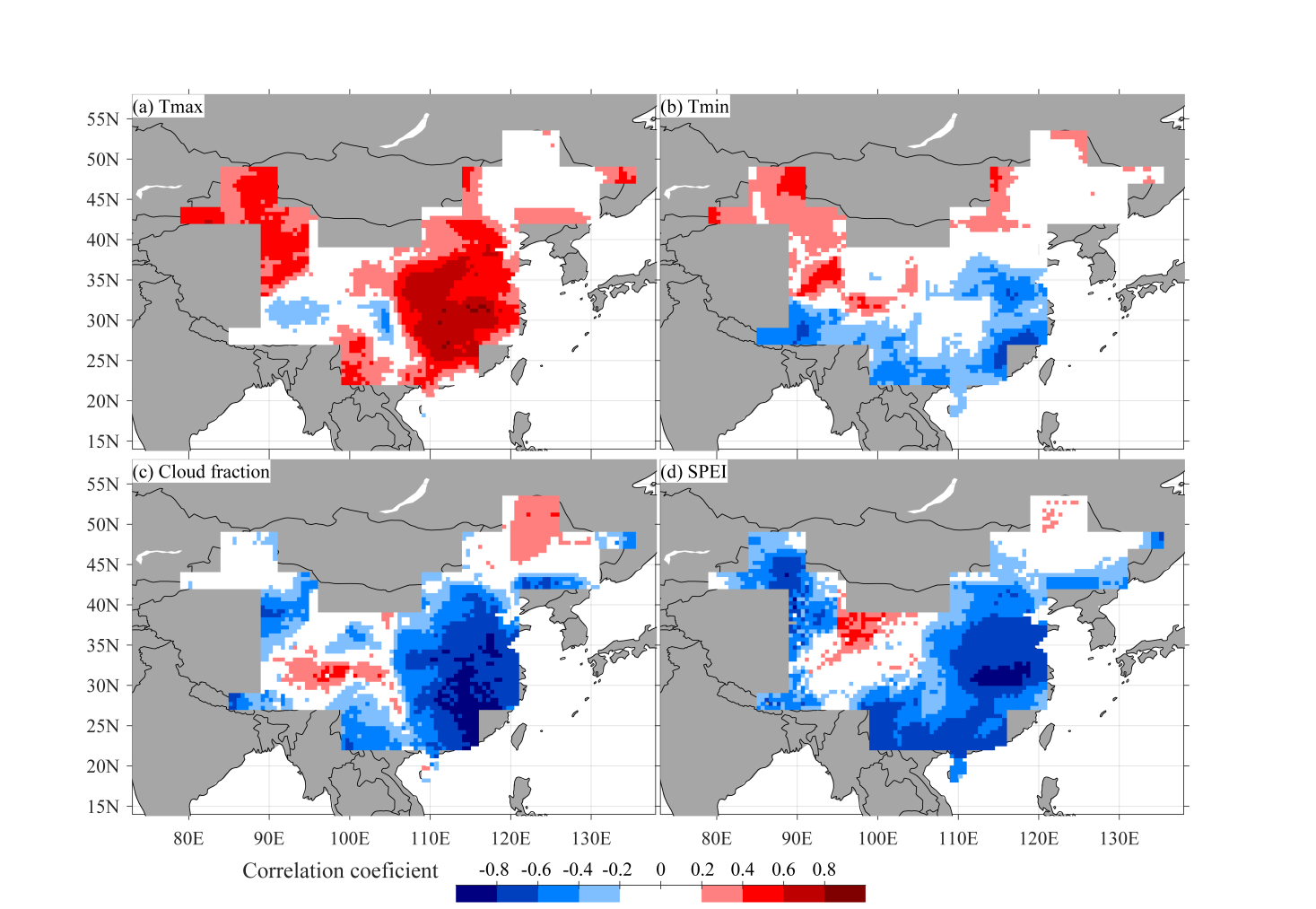


**Supplementary Figure 19**. Pointwise correlations between the unfiltered (not being first differenced) number of gridded fires and the (a) maximum temperature (Tmax), (b) minimum temperature (Tmin), (c) cloud fraction and (d) Standardised Precipitation-Evapotranspiration Index (SPEI) in the post-monsoon season from October to December. The maximum and minimum temperatures and cloud fraction data were derived into the 0.5°×0.5° gridded Climate Research Unit dataset (CRU TS4.03) data spanning from 1901 to 2018 ^3^. The SPEI is a multiple-timescale drought index ^4^ and were calculated from the CRU data with a 0.5°× 0.5° spatial resolution.


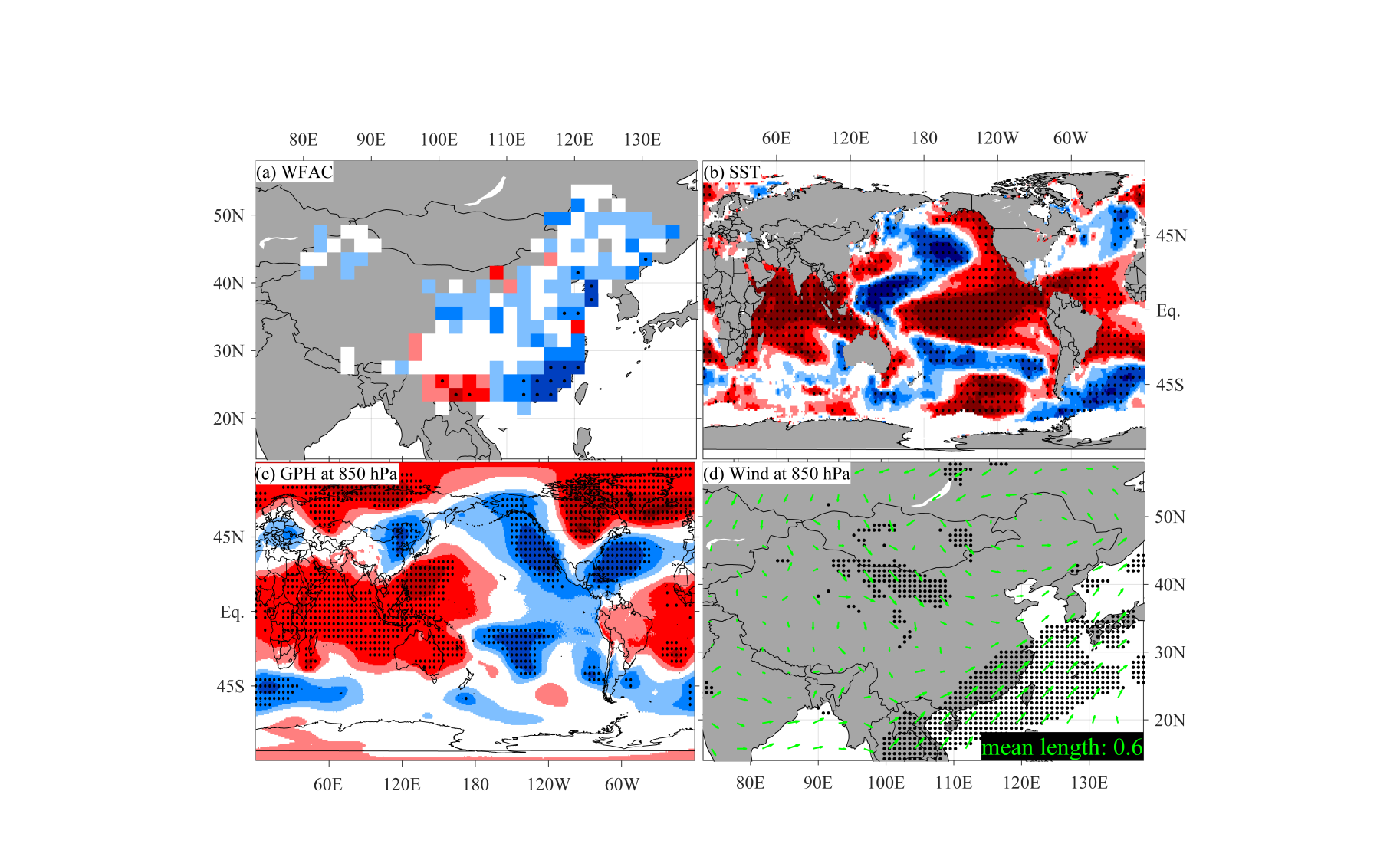


**Supplementary Figure 20**. The same as Fig. 5 in the main text, except for the use of unfiltered (not being first differenced) data.


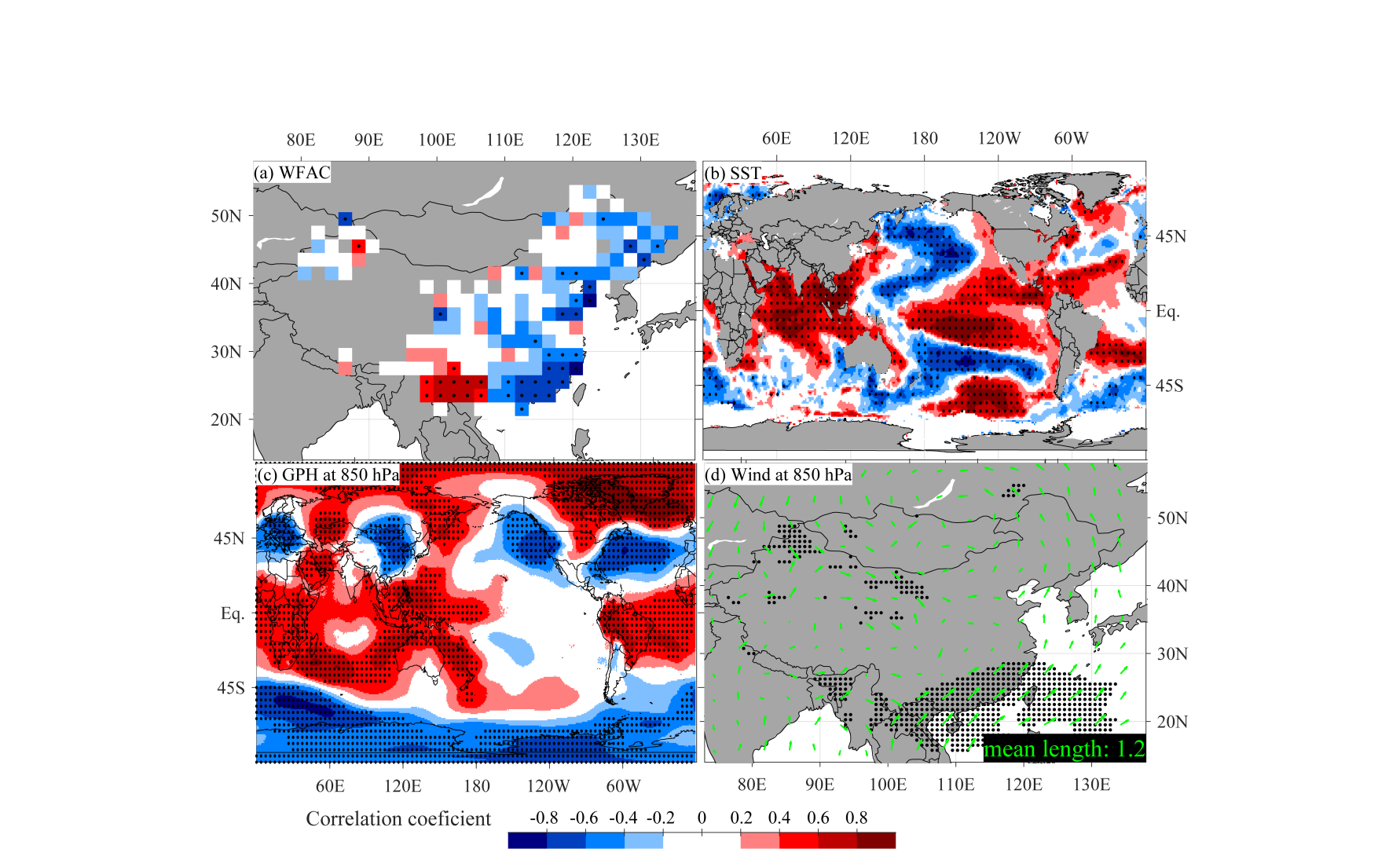


**Supplementary Figure 21.** The same Fig. 5 in the main text, except for the first differenced annual data.


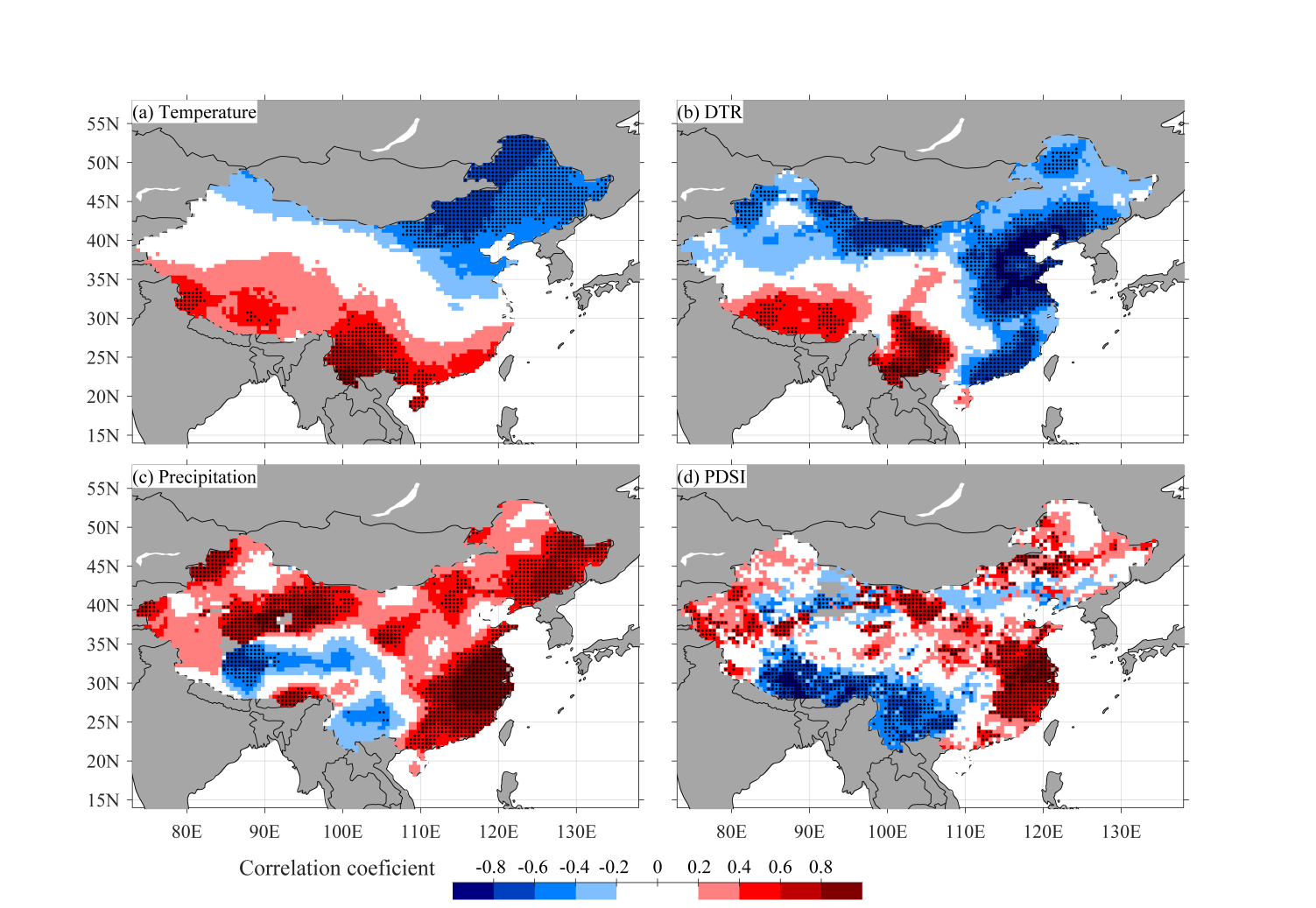


**Supplementary Figure 22.** Correlations between the time series of the first leading singular value decomposition (SVD) between Wildfire Atlas of China (WFAC) and sea surface temperature (SST) and (a) temperature, (b) diurnal temperature range (DTR), (c) precipitation and (d) Palmer drought severity index (PDSI) in the main fire season from January to April for the first differenced data. Regions where the correlation is significant at the 0.05 level are illustrated by black dots. The first differenced data are by calculated as the differences between data of two successive years.

**Supplementary Table 1**. Information on the responses of regional wildfires to the El Niño provided in previous studies.^5^

| Continent | Region/Country | Sign of response to El Niño | References |
| --- | --- | --- | --- |
| Africa | northern Africa | positive | ^5^ |
| Africa | southern Africa | negative | ^5^ |
| Asia | northeastern China | positive | ^6^ |
| Asia | southeastern Asia | positive | [^7^](#_ENREF_3)^,^ [^8^](#_ENREF_4) |
| North America | southwestern USA | negative | [^9^](#_ENREF_5)^,^ [^10^](#_ENREF_6) |
| North America | Pacific northwest | positive | ^11^ |
| South America | Argentina | negative | ^12^ |
| South America | Brazil and Bolivia | positive | ^13^ |
| Oceania | Australia | positive | [^14^](#_ENREF_10)^,^ [^15^](#_ENREF_11) |

**Supplementary Table 2**. Parameters of the polar orbit satellites

| Satellite series | Satellite-borne instruments | Sub-satellite point resolution (m) | NO. spectral channels | Downstream bandwidth (m) |
| --- | --- | --- | --- | --- |
| NOAA | AVHRR | 1,100 | 5 | 2.5 |
| EOS | MODIS | 250/500/1,100 | 36/17 | 30 |
| FY3 | VIRRS | 1,100 | 10 | 6.8 |
|  | MERSI | 250/500/1,100 | 20 | 37.4 |
| METOP | AVHRR | 1,100 | 5 | 30 |
| NPP | VIIRS | 375/750 | 22/18 | 30 |
| JPSS | VIIRS | 400/800 | 16 | 23-183 |

**Supplementary References**

1. Giglio, L., Schroeder, W. & Justice, C. O. The collection 6 MODIS active fire detection algorithm and fire products. *Remote Sens. Environ.* **178,** 31-41 (2016).

2. Chen, J., Yi, F. B. & Song, N. L. China: Open access to Earth land-cover map. *Nature* **514,** 434 (2015).

3. Harris, I., Jones, P., Osborn, T. & Lister, D. Updated high‐resolution grids of monthly climatic observations–the CRU TS3. 10 Dataset. *Int*. *J. Climatol.* **34,** 623-642 (2014).

4. Vicente-Serrano, S. M., Beguería, S. & LópezMoreno, J. I. A Multiscalar Drought Index Sensitive to Global Warming: The Standardized Precipitation Evapotranspiration Index. *J. Clim.* **23,** 1696-1718 (2010).

5. Andela, N., Van, D. W. & Guido, R. Recent trends in African fires driven by cropland expansion and El Nio to La Nia transition. *Nat. Clim. Change.* **4**, 791-795(2014).

6. Yao, Q. et al. Pacific-Atlantic Ocean influence on wildfires in northeast China (1774 to 2010). *Geophys*. *Res*. *Lett*. **44,** 1025-1033 (2017).

7. Cochrane, M. A. Fire science for rainforests. *Nature* **421,** 913-919(2003).

8. Chen, Y. et al. A pan-tropical cascade of fire driven by El Niño/Southern Oscillation. *Nat*. *Clim*. *Chang*. **7,** 906-911 (2017).

9. Swetnam, T. W. & Betancourt, J. L. Fire-Southern Oscillation Relations in the Southwestern United States. *Ence*. **249,** 1017-1020(1990).

10. Trouet, V., Taylor, A. H., Wahl, E. R., Skinner, C. N. & Stephens, S. L. Fire-climate interactions in the American West since 1400 CE. *Geophys. Res. Lett*. **37,** L04702(2010).

11. Heyerdahl, E. K., McKenzie, D., Daniels, L.D., Hessl, A. E., Littell, J. S. & Mantua, N. J. Climate drivers of regionally synchronous fires in the inland Northwest(1651-1900). *Int. J. Wildland Fire*. **17,** 40-49(2008).

12. Kitzberger, T. & Veblen, T. T. Influences of humans and ENSO on fire history of Austrocedrus chilensis woodlands in northern Patagonia, Argentina. *Ecoscience* **4,** 508-520(1997).

13. Chen, Y. et al. Forecasting fire season severity in South America using sea surface temperature anomalies. *Science* **334,** 787-791 (2011).

14. King, A. D., Pitman, A. J., Henley, B. J., Ukkola, A. M. & Brown, J. R. The role of climate variability in Australian drought. *Nat*. *Clim*. *Chang*. **10,** 177-179 (2020).

15. Mariani, M., Fletcher, M. S., Holz, A. & Nyman, P. ENSO controls interannual fire activity in southeast Australia. *Geophys. Res. Lett.* **43,**(2016).
