# Supplementary material for: ENSO modulates wildfire activity in China
Source: Nat Commun. 2021 Mar 19;12:1764. doi: 10.1038/s41467-021-21988-6 (PMC7979797; doi:10.1038/s41467-021-21988-6)
Supplement: Supplementary file 3 — Descriptions of Additional Supplementary Files [file 41467_2021_21988_MOESM3_ESM.pdf]

Descriptions of Additional Supplementary Files

Supplementary Data 1

Description: The Wildfire Atlas of China (WFAC) dataset. The dataset is about the number of fire occurrences in a monthly resolution 2005 to 2018.

Supplementary Movie 1

Description: Movie of the monthly number of fire occurrences of the Wildfire Atlas of China (WFAC).
