# Supplementary figures and images for: ENSO modulates wildfire activity in China
Source: Nat Commun. 2021 Mar 19;12:1764. doi: 10.1038/s41467-021-21988-6 (PMC7979797; doi:10.1038/s41467-021-21988-6)

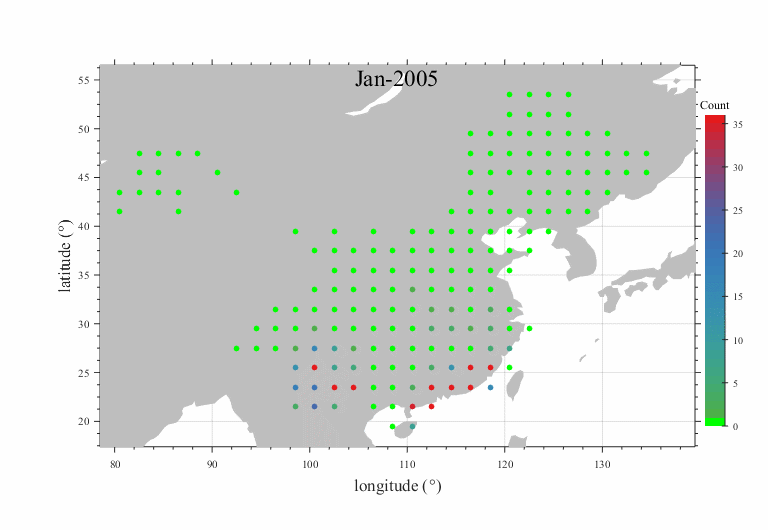

Supplement: Supplementary file 4 — Supplementary Movie 1 [file 41467_2021_21988_MOESM4_ESM.gif]
